# Transfusion Requirements for Severe Anemia in Acute Cardiovascular Patients—A Single Center Retrospective Study in Constanta County Cardiology Department

**DOI:** 10.3390/jcm13237235

**Published:** 2024-11-28

**Authors:** Sevigean Ali, Iulia Andreea Badea, Mihaela Botnarciuc, Lavinia Carmen Daba, Andreea Alexandru, Liliana-Ana Tuta, Irinel Raluca Parepa, Alina Mihaela Stanigut, Mihaela Ionescu

**Affiliations:** 1 Preclinical Disciplines Department, Faculty of Medicine, Campus B, Ovidius University of Constanta, Aleea Universitatii nr. 1, 900470 Constanta, Romania; sevigean.ali@365.univ-ovidius.ro (S.A.); iulia.badea@365.univ-ovidius.ro (I.A.B.); mihaela.botnarciuc@univ-ovidius.ro (M.B.); lavinia.daba@univ-ovidius.ro (L.C.D.); 2 Blood Transfusions Unit, Emergency Clinical County Hospital Constanta, Bdul Tomis nr. 145, 900591 Constanta, Romania; 3 Nephrology Department, Emergency Clinical County Hospital Constanta, Bdul Tomis nr. 145, 900591 Constanta, Romania; alexandra_med16@yahoo.com; 4 Clinical Medical Disciplines Department, Faculty of Medicine, Campus B, Ovidius University of Constanta, Aleea Universitatii nr. 1, 900470 Constanta, Romania; asburlan29@yahoo.com; 5 Cardiology Department, Emergency Clinical County Hospital Constanta, Bdul Tomis nr. 145, 900591 Constanta, Romania; ciucea_mihaela@yahoo.com

**Keywords:** anemia, blood transfusions, heart failure, chronic kidney disease, mortality

## Abstract

**Background:** Anemia is common in hospitalized cardiac patients and affects prognosis and cardiovascular mortality in patients with acute decompensated heart failure. *Aim:* to investigate the impact of anemia severity, blood transfusion practices, and the evolution and outcome in patients with acute cardiovascular events. **Methods:** We performed a retrospective analysis of the patients hospitalized in the Cardiology Department of Constanta County Hospital who required blood derivatives transfusions, between 1 January 2021 and 31 December 2021. **Results:** Out of the total 270 patients, 170 received a single unit of resuspended erythrocyte concentrate within the same month, while 100 required multiple transfusions, receiving between 2 and 5 units during a single hospitalization, to correct anemia. Before transfusions, the mean hemoglobin (Hb) level was 7.60 g/dL, with values ranging from 6.50 g/dL to 9.10 g/dL. Men show a higher prevalence (64%) than women (36%), likely due to gender differences in susceptibility to heart conditions. Patients with associated acute or chronic renal failure consistently experience higher in-hospital mortality in all left ventricular ejection fraction (LVEF) subgroups. **Conclusions:** Anemia in heart failure patients is linked to worsening symptoms, decreased kidney function, and higher hospitalization and mortality rates. The findings aim to inform and optimize clinical decision making, particularly regarding transfusion strategies and risk management in this high-risk population.

## 1. Introduction

Heart failure (HF) is a pathological condition with a continuously increasing prevalence, associated with significant mortality and morbidity defined by the heart’s inability to provide sufficient systemic blood flow to meet the body’s energy needs [1]. In the global context of an epidemic of inappropriate lifestyle diseases, like obesity, diabetes mellitus, and coronary artery disease, it is estimated that globally the number of HF patients will increase by 25% by the year 2030 [2]. Despite the new therapeutical discoveries in the fields of medical and device therapies, the mortality rate of HF patients remains high, at about 50% at 5 years of diagnosis, especially in patients with reduced ejection fraction (HFrEF) [3]. For patients with HF, the primary management recommendation is the treatment of the underlying cardiac disease, but also to address comorbidities to improve prognosis.

Anemia is one of the most frequently encountered comorbidities detected in cardiac hospitalized patients, and almost 50% of these patients have iron deficiency (ID), with unfavorable prognostic consequences. The prevalence of anemia in patients with HF varies between 10% and 50%, depending on the population studied, the functional class of heart failure, and the criteria used for diagnostics. Patients with congestive heart failure refractory to medical treatment are more frequently anemic. The prevalence of anemia in this group of patients approaches 80%, while in stable patients, with functional class I or II (NYHA), it is less than 10% [4].

Anemia in patients with HF leads to a worse cardiac and functional status compared to those without anemia. Although the cause of anemia in HF is not entirely clear, evidence suggests that neurohormonal and proinflammatory cytokine activation and renal dysfunction favor the development of anemia of chronic kidney disease (CKD) [5]. Although erythropoietin stimulating agents (ESAs) were considered a rational therapy to increase hemoglobin and to treat anemia in CKD patients with HF, these agents do not improve outcomes and may be associated with thromboembolic complications. ESAs are therefore not recommended [5]. While treatment of iron deficiency demonstrated evident symptomatic improvement in HF patients, correction of anemia using blood transfusions has failed to show any significant positive outcomes. But there are still controversies regarding the correction of severe anemia (Hb < 8 g/dL) in symptomatic patients with severe HF [6].

In the prospective STAMINA-HFP study (Study of Anemia in a Heart Failure Population), the prevalence of anemia in HF was 34%, and a meta-analysis conducted in patients with HF showed a prevalence of anemic syndrome of 37.2% [7]. Patients with chronic kidney disease (CKD) and HF develop anemia with a higher glomerular filtration rate (GFR) compared to patients with CKD without HF. This supports the hypothesis that other factors beyond renal dysfunction are associated with anemia in patients with HF [8,9].

Cardiorenal syndrome (CRS) represents a complex interplay between cardiac and renal dysfunction, where the impairment of one organ adversely affects the other. This bidirectional relationship is particularly evident in patients suffering from CKD and anemia, as these conditions often co-occur and exacerbate one another [10].

The Consensus Conference of the Acute Dialysis Quality Initiative (ADQI) classifies cardiorenal syndrome (CRS) into five types based on the interaction and impact between cardiac and renal dysfunction:

Type 1: Acute Cardiorenal Syndrome—Acute worsening of heart function leads to acute kidney injury (e.g., acute heart failure causing AKI).

Type 2: Chronic Cardiorenal Syndrome—Chronic heart disease causes chronic kidney disease (e.g., chronic heart failure progressively impairing renal function).

Type 3: Acute Renocardiac Syndrome—Acute kidney injury triggers acute heart dysfunction (e.g., AKI resulting in acute heart failure).

Type 4: Chronic Renocardiac Syndrome—Chronic kidney disease (CKD) promotes chronic heart dysfunction (e.g., CKD contributing to chronic heart failure).

Type 5: Secondary Cardiorenal Syndrome—Systemic conditions cause simultaneous heart and kidney dysfunction (e.g., sepsis or diabetes affecting both organs).

This classification emphasizes the complex, bidirectional interactions between the heart and kidneys, highlighting the need for coordinated, targeted treatment approaches tailored to the specific type of CRS [11].

The pathophysiology of CRS is multifactorial, involving hemodynamic changes, neurohormonal activation, and systemic inflammation. For instance, reduced cardiac output can lead to impaired renal perfusion, while renal dysfunction may result in fluid overload and increased cardiac workload. Furthermore, the overactivation of the Renin–Angiotensin–Aldosterone System (RAAS) plays a crucial role in the progression of both cardiac and renal diseases, contributing to the worsening of anemia through mechanisms such as erythropoietin resistance [11,12].

## 2. Aim

This study aimed to investigate the impact of anemia severity and blood transfusion practices, and the evolution and outcome in patients with acute cardiovascular events. By examining the relationships between anemia severity, transfusion frequency, occurrence of alloimmunization, and patient outcomes, this study aims to provide a comprehensive understanding of prognostic factors and potential complications in patients with acute, congestive heart failure.

The findings aim to inform and optimize clinical decision making, particularly regarding transfusion strategies and risk management of alloimmunization in this high-risk population.

## 3. Materials and Methods

We performed a retrospective analysis of the patients hospitalized in the Cardiology Department of Constanta County Hospital for congestive heart failure (CHF) and variable degrees of kidney insufficiency who required blood derivative transfusions between 1 January 2021–31 December 2021.

Inclusion criteria were adults aged ≥ 18 years, capable of understanding and providing written informed consent (or provision by a legal representative), diagnosed with different degrees of anemia, with confirmed diagnosis of acute heart failure, based on established clinical guidelines (e.g., European Society of Cardiology [ESC] or American Heart Association [AHA] criteria), including symptoms like dyspnea, fluid overload, and evidence of structural or functional cardiac abnormalities.

Exclusion criteria: Patients with active bleeding at the time of admission, known clotting disorders, recent therapeutic anticoagulation treatment, a history of organ or bone marrow transplant, severe infections at admission, or primary hematologic or oncologic diseases.

The exposure variables were collected during hospital admission as follows: (a) demographic characteristics (sex, age, medication); (b) NYHA class (III, IV, LVEF); (c) renal function (eGFR, serum creatinine, urea); (d) main laboratory data (transferrin saturation, ferritin, iron, hemoglobin, hematocrit); and (e) number of blood derivatives transfused per patient and per year.

The diagnosis of heart failure was established using the diagnostic algorithm developed by the European Society of Cardiology [3]. The algorithm involves a pretest that assesses for HF symptoms and signs, clinical demographic findings (obesity, hypertension, diabetes, elderly), and diagnostic laboratory tests, electrocardiogram (ECG), and echocardiography. In the absence of overt noncardiac causes of breathlessness, heart failure with preserved ejection fraction (HFpEF) could be suspected if there is a normal LVEF, no significant heart valve disease, or cardiac ischemia, and at least 1 typical risk factor. The score uses functional, morphological, and biomarker domains. The points score assigns 2 points for a major criterion or 1 point for a minor criterion within each domain, with a maximum of 2 points for each domain.

The complete blood count was performed on the Sysmex XN 1000 analyzer (flow cytometry with hydrodynamic focus). Each patient was tested for the blood group in the ABO system, as well as the Rh D factor and Rh phenotype. Administration of ABO-matched and Rh-phenotype-matched resuspended erythrocyte concentrate was intended to enhance transfusion efficiency and reduce the risk of immunization. Alloimmunization was tested by detecting irregular antibodies (IATs) by the gel column agglutination method using Low Ionic Strength Saline/Coombs and Enzyme cards.

Creatinine levels were monitored dynamically, with the estimation of glomerular filtration rate (eGFR) and urinary output, to diagnose patients with renal failure. Some of the patients, with previous normal kidney function before admission, were diagnosed with acute kidney injury (AKI) based on widely accepted criteria such as the KDIGO (Kidney Disease: Improving Global Outcomes) classification, defined by increases in serum creatinine and/or decreased urine output over a specified time period (7 days). Other patients were already diagnosed with variable grades of chronic kidney disease (CKD- KDIGO 2012 Classification), defined by a glomerular filtration rate (GFR) < 60 mL/min/1.73 m^2^, albumin-to-creatinine ratio > 30 mg/g creatinine, or markers of kidney damage (e.g., hematuria or structural abnormalities such as hypoplastic, polycystic, or dysplastic kidneys, and morphopathologically specific abnormalities in kidney biopsy) persisting for more than 3 months.

A comprehensive statistical analysis was performed to evaluate the differences between clinical and laboratory parameters across study groups. The normality of continuous variables was first assessed using the Shapiro–Wilk test, and due to the non-Gaussian distribution of most variables, non-parametric methods were applied. The Mann–Whitney U test was used to compare the medians of continuous variables between two independent groups, as this test does not require the assumption of normal distribution. Categorical variables were summarized as frequencies and percentages and analyzed using the chi-square test to assess significant differences between groups. For categorical data with low expected cell counts (<5), Fisher’s exact test was used to ensure valid results.

Additionally, contingency tables were built to explore relationships between categorical variables, such as the risk of cardiovascular events in patients with and without CKD. Odds ratios (OR) and 95% confidence intervals (CIs) were calculated to quantify the strength of associations. Statistical significance was set at a *p*-value of <0.05 for all analyses, and exact *p*-values were reported to ensure clarity and precision in the findings. This multi-faceted approach, incorporating non-parametric tests and contingency analysis, provided a robust framework for examining the trial’s clinical outcomes, ensuring appropriate handling of non-Gaussian data and categorical variables. All analyses were conducted using GraphPad Prism 8.4.3, ensuring accuracy and reproducibility of results.

## 4. Results

Out of the total 270 patients hospitalized for congestive heart failure, 170 received a single unit of resuspended erythrocyte concentrate, while 100 required multiple transfusions, receiving between 2 and 5 units during a single hospitalization, for correction of severe anemia (Table 1). In percentages, 62% of patients were single-transfused, and 38% were multi-transfused.

Table 2 illustrates the distribution of male and female patients across various age groups, highlighting how gender composition varies with age.

The patients’ distribution based on diagnosis and gender highlighted significant differences between males and females for each analyzed cardiac condition. Our data suggest that while men have a higher prevalence of many acute cardiac conditions, women are not excluded from the risks associated with cardiovascular diseases.

Heart failure is a significant health concern within this patient population, indicating the need for targeted management strategies to address the complexities associated with this condition. We analyzed the prevalence of various cardiac conditions, admitted via ER due to their manifestations of acute heart failure. The highest percentage of our hospitalized patients (32.6%) were diagnosed with arrhythmias (atrial fibrillation, atrial flutter).

We noticed that ST elevation myocardial infarction (STEMI) accounted for 30% of the cases, while the other acute coronary syndromes, non-ST elevation myocardial infarction (NSTEMI) and unstable angina (UA) represented 20%, whereas metabolic cardiomyopathies (diabetes, obesity-related) accounted for 17.4%, as described in Table 3.

Patients with and without chronic kidney disease (CKD) were compared based on clinical and laboratory data (Table 4). Patients are divided into two groups: those who received 1–2 units and those who received 3–4 units of an unidentified treatment. There are statistically significant differences in several variables, indicating important clinical features. Older age (≥65 years) is substantially more common in the 1–2-unit group (65%) than in the 3–4-unit group (43%, *p* = 0.04) among patients without CKD. In the 3–4-unit group, women also make up a greater percentage (71% vs. 30%, *p* = 0.001).

There are noteworthy variations in the severity of heart failure as well. For example, left ventricle ejection fraction (LVEF) was lower in the group of patients in the 3–4-unit group (31% vs. 35%, *p* = 0.001), and NYHA classes III–IV showed considerable variation (*p*-values between 0.03 and 0.05). Data from laboratories demonstrate that hemoglobin, iron, transferrin saturation, and hematocrit are significantly lower in the 3–4-unit group (all with *p*-values < 0.05). Creatinine is slightly elevated in the 3–4-unit group (*p* = 0.03), indicating reduced kidney function.

Patients with and without chronic kidney disease (CKD) are compared in Table 5. Similar trends are seen in CKD patients (Table 5). In the 1–2-unit group, there is a greater proportion of women (42% vs. 29%, *p* = 0.02) and older age (90% vs. 62%, *p* = 0.001). For NYHA classes III and IV, there are significant differences in the severity of heart failure (*p* = 0.02 and *p* = 0.05). The 3–4-unit group has reduced hemoglobin, iron, and transferrin saturation, according to laboratory results (*p*-values between 0.001 and 0.03). The 3–4-unit group has considerably greater hematocrit (*p* = 0.001). Notably, eGFR (*p* = 0.01) and creatinine (*p* = 0.05), two indicators of kidney function, show that the renal function of the 3–4-unit group is lower than that of the 1–2-unit group.

With an OR of 2.07, arrythmias are statistically significantly more common in CKD patients (52 with CKD vs. 36 without CKD, *p* = 0.006), suggesting that CKD patients have a roughly two-fold increased risk of developing arrythmias in comparison to those without CKD. A 2.6-fold increased risk of myocardial infarction (MI) is suggested by the considerably higher frequency of MI in patients with chronic kidney disease (CKD) (56 with vs. 37 without CKD, *p* = 0.005), with an OR of 2.6. An OR of 0.8 for metabolic cardiomyopathies (CMPs) indicates no substantial correlation between CKD and metabolic CMP in this group, with no significant difference observed between CKD and non-CKD patients (24 with CKD vs. 23 without CKD, *p* = 0.6). Conversely, acute coronary syndromes (ACSs), either STEMI or non-STEMI and unstable angina, exhibit a strong and significant association with CKD (42 with CKD vs. 12 without CKD, *p* = 0.001), with an OR of 3.9, indicating that CKD patients are nearly four times more likely to experience ACS compared to those without CKD.

Regarding the distribution of RBC units, the data for the prevalence of single unit transfusions indicate that most patients (62.9%) received only 1 unit of red blood cells. Regarding the decrease in multiple unit transfusions, the percentage of patients receiving 2 units (18.9%), 3 units (11.5%), and 4 units (6.7%) of REC is significantly lower than those receiving a single unit.

CRS type 1 is a significant clinical condition that associates the development of acute kidney injury (AKI) and dysfunction in the patient with acute cardiac illness, most commonly acute decompensated heart failure (ADHF).

In our study, CRS type 1 was found in 40 non-CKD patients (26%); 29 were discharged, recovering previous normal kidney function; and the mortality was 4.4% in this group. We compared the renal outcome and mortality based on hemoglobin level and the transfusions performed. All of them received 2 to 4 blood transfusions units, and we describe the hemodynamic parameters in Table 6.

Median hemoglobin levels rose from 7.3 g/dL (IQR: 6.4–8.8) at admission to 9.8 g/dL (IQR: 7.9–10.2) at discharge, with a statistically significant *p*-value of 0.001, indicating marked hematological improvement. Systolic BP also showed a significant increase from a median of 90 mmHg (IQR: 70–100) at admission to 100 mmHg (IQR: 84–109) at discharge (*p* = 0.03), suggesting stabilization in cardiovascular status. Left ventricular ejection fraction (LVEF) improved from a median of 45% (IQR: 35–50) to 50% (IQR: 46–53) at discharge (*p* = 0.004), indicating enhanced cardiac function. Furthermore, renal function markers showed notable improvement: creatinine levels decreased from a median of 4.76 mg/dL (IQR: 3.54–6.56) at admission to 0.99 mg/dL (IQR: 0.46–1.39) at discharge (*p* = 0.001), while urea levels fell from 137.56 mg/dL (IQR: 109.3–219.23) to 42 mg/dL (IQR: 23.2–58.8) (*p* = 0.03). These statistically significant changes underscore meaningful clinical recovery in multiple organ systems by the time of discharge.

When comparing mortality between the CKD patients and the non-CKD group, including the CRS type 1 group, we found a significant difference (*p* = 0.001, OR = 2.2), indicating that patients with AKI (CRS type 1) have a 2.2 times higher risk of death from multi-organ failure compared to CKD patients (as expressed in Table 7).

We noticed significant differences in in-hospital mortality between patients with CKD, without CKD (non-CKD), and CRS type 1. Among all patients, in-hospital mortality is higher in those with CRS type 1 (22.5%) compared with CKD (16.8%), and those non-CKD with normal kidney function (18.1%), and this difference is significant (*p* = 0.03). In patients with LVEF > 50%, mortality is higher in those with CKD and CRS type 1 (*p* = 0.001), suggesting that a higher LVEF does not sufficiently reduce the risk of mortality for these patients. For patients with LVEF < 50%, in-hospital mortality remains high for both CKD and CRS type 1 patients, being statistically significant (*p* = 0.04), showing that mortality risk is increased by comorbidities such as CKD and AKI, regardless of LVEF.

Regarding distribution of RBC units, and prevalence of single unit transfusions, the data indicate that most of the patients (62.9%) received only 1 unit of red blood cells.

We reported a decreased use of multiple unit transfusions, so the percentage of patients receiving 2 units was 18.9%, 3 units was 11.5%, and 4 units only 6.7%, which are significantly lower than those receiving a single unit, as presented in Table 8.

As emphasized in Table 9, a significant proportion of patients across all diagnostic categories have hemoglobin levels below 8 g/dL, indicating a potential state of severe anemia. Notably, 19.2% of patients with arrythmias (atrial fibrillation or atrial flutter) fall into this category, which is the highest percentage among the diagnoses listed. Comparative analysis shows that, while STEMI also shows a substantial number of patients with Hb levels below 8 g/dL (15.9%), other diagnoses such as acute coronary syndrome (NSTEMI + UA) and metabolic CMP have lower percentages (10.7% and 10.4%, respectively). This variation may reflect differences in underlying pathophysiology or management strategies among these patient groups. The number of patients with hemoglobin levels above 8 g/dL is lower across all categories. The percentages range from 7.1% in arrhythmias to 14.0% in MI. This trend suggests that while some patients maintain higher hemoglobin levels, there is still a notable category at risk of complications related to lower levels of Hb, particularly in the context of their cardiovascular conditions.

## 5. Discussions

Recent studies showed that the prevalence of anemia in patients with HF (defined as hemoglobin <13 g/dL in men and <12 g/dL in women) is detected in about 50% of hospitalized patients, both in those with HFrEF or HFpEF, compared with <10% in the general population. Anemic HF patients are more frequently females, older, diabetics, and those with associated CKD, in comparison to non-anemic HF patients. Anemia aggravates HF, with patients presenting worse functional status, lower exercise capacity, worse quality of life (QoL), lower blood pressure, greater edematous syndrome with higher requirement of diuretics, and an increased neurohormonal and proinflammatory cytokine activation [5].

Our data suggest that while men have a higher prevalence of many acute cardiac conditions, women are not excluded from the risks associated with cardiovascular diseases. As age increases, the proportion of female patients becomes more significant, particularly in the 60–70 and 70–80 decades. This could suggest a longer life expectancy for females or a higher incidence of age-related health issues that affect females more significantly. For male patients, the highest percentage (29%) is found in the 60–70-year age group, followed closely by the 70–80-year group (24%). This indicates a notable representation of males in the older age categories, which may reflect patterns in health risks and disease prevalence in this demographic. In contrast, the female patients show their highest representation in the 70–80-year age group (39%), suggesting that this age group may face specific health challenges that lead to increased healthcare needs.

CRS type 1 is a significant clinical condition that associates the development of acute kidney injury (AKI) and dysfunction in the patient with acute cardiac illness, most commonly acute decompensated heart failure (ADHF). The multiple pathophysiological mechanisms operating concomitantly include acute congestion in both the heart and kidneys, neurohormonal activation, immune cell and cytokine signaling disruptions, superimposed infections, anemia, and the breakdown of normal counter-regulatory mechanisms. Together, these factors drive a worsening cycle of cardiac and renal dysfunction [12].

Our findings emphasize that older age, high NYHA class, anemia indicators, and renal function are key differentiators in patients receiving different levels of treatment, both with and without CKD, confirming data from relevant studies [13,14,15,16]. On the other hand, CHF associated with arrhythmias, STEMI, and ACS (NSTEMI + UA) are significantly more common in patients with CKD, as evidenced by low *p*-values (<0.01) and high odds ratios, whereas metabolic CMP, paradoxically, does not show a significant association. This suggests that CKD is a strong predictor of adverse cardiovascular outcomes, particularly CHF due to arrhythmias, MI, and ACS, but not metabolic cardiomyopathies (diabetes, obesity, and alcohol abuse) in this cohort, which does not match with reported data [17]. Our findings indicate that CKD patients consistently experience higher in-hospital mortality across LVEF subgroups, while CRS patients have relatively lower mortality rates, with significant associations observed between reduced LVEF and increased mortality across all patient groups. Also, our findings suggest that arrythmia patients may be more prone to developing anemia, potentially due to chronic disease factors or comorbidities.

The significant prevalence of low hemoglobin levels, especially in acute HF, underscores the importance of monitoring and addressing anemia in patients with cardiovascular diseases. Anemia can exacerbate heart failure symptoms and negatively impact overall prognosis, as noticed in cardiac surgery patients [18,19,20]. Therefore, timely interventions, including iron supplementation or blood transfusions, may be necessary to improve patient outcomes.

Regarding transfusion treatment, our results indicates that most patients (62.9%) received only 1 unit of red blood cells. This may reflect a practice of restrictive transfusion strategies aimed at minimizing the risk of transfusion-related complications. Also, it suggests that most cases did not require aggressive transfusion interventions, which is consistent with current guidelines recommending minimal transfusions when possible [18,21,22]. Additionally, the correlation between the number of blood units transfused and mortality in CRS patients showed that prognosis worsens as the number of transfused units increases.

Regarding healthcare costs, it is obvious that using single units may also have cost implications, as transfusion-related resources, including donor blood supply and monitoring requirements, could be minimized. This is beneficial for healthcare systems aiming to optimize resource allocation.

While packed RBC transfusions can serve as short-term therapy, they carry significant risks and offer only temporary relief. Kao et al. analyzed data from a large public discharge database encompassing 596, 456 patients admitted with heart failure (HF), finding anemia in 27% of cases. Untreated anemia was linked to a roughly 10% higher adjusted mortality risk, but in anemic HF patients receiving transfusions, this risk rose to approximately 70%. These findings raise concerns about potential harm from transfusions in HF patients; however, key limitations exist in the database analysis, including the lack of data on anemia severity and specific clinical indications for transfusion, which were not accounted for. These factors, along with other residual confounders, may have influenced the multivariable analysis results [23].

We decided not to apply a multivariate regression because the model would require a larger sample to provide robust and valid results, and currently the small number of observations limits the ability to identify significant effects. In addition, the variables analyzed exhibited multicollinearity, which may lead to unreliable estimates and misinterpretations of coefficients. In this context, the individual interpretation of the effects of the risk variables is more appropriate and allows clearer conclusions to be drawn for this stage of the research.

Prospective randomized controlled trials (RCTs) are needed to clarify the role of RBC transfusions in patients with HF and anemia. Notably, the TRICS III trial, which involved moderate- to high-risk cardiac surgery patients, demonstrated no significant difference in the composite outcome (death, MI, stroke, or new-onset renal failure with dialysis) between restrictive transfusion (hemoglobin < 7.5 g/dL) and a more liberal transfusion threshold (hemoglobin < 9.5 g/dL), suggesting that a restrictive approach is noninferior in such patients [19]. These findings imply that RBC transfusions in anemic HF patients may not always yield a benefit and could be linked to adverse outcomes. Consequently, routine transfusion in asymptomatic patients, especially those with stable anemia, cannot be recommended. Given the variation in hemoglobin thresholds for transfusion across clinical guidelines, individual factors such as age, comorbidities, and surgical requirements should be carefully considered when determining transfusion needs in HF patients [24,25].

The main limitation of this study is its reliance on registry data, introducing potential confounders from uncontrolled variables, such as iron levels and history of chronic anemia. As a sub-analysis of an observational study, it is also subject to unmeasured confounders typical of such designs. Additionally, the reasons for underutilization of medications or procedures remain unknown. Optional recording of natriuretic peptides and variable echocardiographic interpretation, without centralized review, may have influenced the results. Data on renal function at discharge and recovery frequency are unavailable for the entire group, as are hydro-electrolytic and acid–base disturbances, which significantly impact morbidity, mortality, and 1-year outcomes. Mortality follow-up was limited to a single year, without specific dates of death, precluding Kaplan–Meier analysis. The study also lacks information on stroke etiology. Future studies should address these limitations to enhance validity.

## 6. Conclusions

Our study reveals that patients requiring higher transfusion volumes (3–4 units) exhibit more severe HF (e.g., lower LVEF, higher NYHA class), regardless of the renal function status; they also present with more severe anemia (lower hemoglobin, iron, transferrin saturation, and hematocrit levels). Older patients more frequently required 1–2 units, especially among non-CKD individuals, while elevated creatinine and reduced eGFR were prevalent in the 3–4-unit group across both CKD and non-CKD patients, linking kidney dysfunction to higher transfusion demands.

In patients with CRS type 1, characterized by acute HF with multi-system complications, in-hospital mortality was 4.4%, with notable post-transfusion improvements in hemoglobin, systolic BP, LVEF, and renal markers by discharge, suggesting hematological and cardiovascular recovery. CRS patients faced a 2.2 times higher mortality risk than CKD-only patients, especially from multi-organ failure. Mortality analysis revealed that CKD patients had the highest mortality, even with preserved LVEF, while CRS patients had better in-hospital outcomes, despite transfusion volume correlating with poorer prognosis in CRS. These findings underscore the complex interplay of heart failure severity, renal impairment, and anemia in determining outcomes in CRS and CKD.

## Figures and Tables

**Table 1:** Distribution of patients based on the number of received RBC units/month.

Month	2021	1 Unit Transfused	2 or More Units Transfused
January	25	12	13
February	28	17	11
March	24	19	5
April	19	16	3
May	23	16	7
June	14	6	8
July	15	12	3
August	24	17	7
September	28	15	13
October	22	12	10
November	27	18	9
December	21	10	11
Total	270	170	100

**Table 2:** Distribution based on age and gender.

Gender	<50 Years	50–60 Years	60–70 Years	70–80 Years	≥80 Years
Male (n = 173)	7 (4%)	31 (18%)	50 (29%)	42 (24%)	43 (25%)
Female (n = 97)	0%	6 (6%)	30 (31%)	38 (39%)	23 (24%)

**Table 3:** Distribution based on causes of CHF.

Diagnosis	Number	Percentage
STEMI	81	30%
NSTEMI + UA	54	20%
ARRHYTHMIAS	88	32.6%
METABOLIC CARDIOMYOPATHY (CMP)	47	17.4%

**Table 4:** Variables analyzed in CKD and non-CKD patients associated with blood transfusions.

Variables	Total (n = 270)	Non-CKD (n = 116)			CKD (n = 154)		
		**1–2 Units** **(n = 109)**	**3–4 Units** **(n = 7)**	***p*-Value**	**1–2 Units** **(n = 112)**	**3–4 Units** **(n = 42)**	***p*-Value**
**Age (>65 yrs)**	201 (74%)	71 (65%)	3 (43%)	0.04	101 (90%)	26 (62%)	0.001
**Women**	97 (36%)	33 (30%)	5 (71%)	0.001	47 (42%)	12 (29%)	0.02
	**NYHA class**						
III	139 (51%)	41 (38%)	5 (72%)	0.05	66 (59%)	27 (64%)	0.02
IV	131 (49%)	36 (33%)	5 (71%)	0.04	66 (59%)	24 (58%)	0.05
**LVEF**	38% (28–43)	35% (30–36)	31% (28–32)	0.001	36 (30–38)	31 (28–37)	0.12
	**Laboratory findings**						
**Transferrin saturation**	18.9 (14.2–26.7)	32.1 (22.3–42.8)	21.7 (18.2–0.4)	0.001	23.4 (20.8–29.4)	18.3 (14.8–21.9)	0.001
**Ferritin**	106 (57–201)	219 (192–274)	198 (177–229)	0.05	158 (130–172)	50 (41–67)	0.07
**Iron**	48 (22–83)	142 (131–158)	122 (109–138)	0.001	95 (87–103)	67 (57–71)	0.03
**Hemoglobin**	7.7 (7.5–9.3)	8.2 (7.3–9.1)	7.4 (6.3–8.1)	0.02	7.6 (6.7–8.0)	6.3 (5.9–6.9)	0.001
**Hematocrit**	32.5	36.5% (35.8–37.2)	41.7 (40.9–2.5)	0.02	44.3 (43.7–44.9)	48.9 (48.2–49.6)	0.001
	**Renal** **Function**						
**eGFR**	76 (43–94)	110.9 (108.4–13.4)	95.6 (93.2–98)	0.08	78.3 (76.5–80.1)	62.4 (61.0–63.8)	0.01
**Creatinine**	2.3 (1.7–2.9)	1.5 (1.3–1.7)	2.2 (2–2.4)	0.03	3.2 (2.7–3.4)	3.8 (3.5–4.1)	0.05
**Urea**	83 (45.4–123.7)	20 (18–24)	35 (32–39)	0.09	50 (46–58)	73 (61–82)	0.08
	**Medication**						
**Antiplatelets**	189 (70%)	87 (80%)	2 (29%)	0.23	85 (76%)	15 (36%)	0.15
**Beta-blockers**	237 (88%)	97 (89%)	3 (43%)	0.12	109 (97%)	28 (67%)	0.03
**ACE/ARB**	149 (55%)	54 (50%)	4 (57%)	0.03	65 (65%)	26 (62%)	0.18
**Loop diuretic**	224 (83%)	89 (82%)	5 (71%)	0.8	99 (88%)	31 (74%)	0.26

**Table 5:** Distribution of CKD and non-CKD patients with AHF, according to cardiac primary disease.

Parameter	Total n = 270	CKD (n = 154)	Non-CKD (n = 116)	*p*-Value	OR
ARRYTHMIAS	88 (32.5%)	52 (33.7%)	36 (31%)	**0.006**	2.07
STEMI	81 (30%)	56 (36.3%)	87 (75%)	**0.005**	2.6
NSTEMI + UA	54 (20%)	42 (27.2%)	12 (10.3%)	**0.001**	3.9
METABOLIC CMP	47 (17.4%)	24 (15%)	23 (19.8%)	0.6	0.8

**Table 6:** Comparison of main parameters, admission, and discharge in patients with CRS type 1.

Parameter	Median Value at Admission	Median Value at Discharge	*p*-Value
Hemoglobin (g/dL)	7.3 (6.4–8.8)	9.8 (7.9–10.2)	0.001
Systolic blood pressure (BP)	90 (70–100)	100 (84–109)	0.03
LVEF	45% (35–50)	50% (46%–53%)	0.004
Creatinine (mg/dL)	4.76 (3.54–6.56)	0.99 (0.46–1.39)	0.001
Urea (mg/dL)	137.56 (109.3–219.23)	42 (23.2–58.8)	0.03

**Table 7:** In-hospital mortality in our study group depending on LVEF.

Mortality		CKD (n = 154)	Non-CKD (n = 116)		*p*-Value
			Total Deaths	CRS Type 1 (n = 40)	
In-hospital	All patients	26 (16.8%)	21 (18.1%)	9 (22.5%)	0.03
	LVEF > 50%	8 (5.2%)	4 (3.4%)	3 (7.5%)	0.001
	LVEF < 50%	18 (11.6%)	21 (18.1%)	6 (15%)	0.04

**Table 8:** Distribution based on the number of transfused units.

RBC Units	Number	Percentage
1 unit of RBC	170	62.9%
2 units of RBC	51	18.9%
3 units of RBC	31	11.5%
4 units of RBC	18	6.7%

**Table 9:** Distribution based on Hb levels (g/dL) and the disease-causing CHF.

Hb(g/dL)	STEMI	NSTEMI + UA	METABOLIC CMP	ARRHYTHMIAS
<8 g/dL	43 (15.9%)	29 (10.7%)	28 (10.4%)	52 (19.2%)
>8 g/dL	38 (14%)	25 (9.3%)	19 (7.1%)	36 (13.4%)

## Data Availability

The data presented in this study are available on request from the corresponding author.

## List of Abbreviations

ACE	Angiotensin-Converting Enzyme
ACS	Acute coronary syndrome
AF	Atrial Fibrillation
AKI	Acute Kidney Injury
AHF	Acute Heart Failure
CKD	Chronic kidney disease
CRS	Cardio-Renal Syndrome
EPO	Erythropoietin
Hb	Hemoglobin
HFpEF	heart failure with preserved ejection fraction
HFrEF	heart failure with reduced ejection fraction
LVEF	left ventricle ejection fraction
STEMI	ST elevation myocardial Infarction
NSTEMI	Non- ST elevation myocardial Infarction
NYHA	New York Heart Association
RBC	Red Blood Cells
SRE	Reticuloendothelial System
TNFα	Tumor Necrosis Factor Alpha
UA	Unstable angina
